# The Fate of Spermatogonial Stem Cells in the Cryptorchid Testes of RXFP2 Deficient Mice

**DOI:** 10.1371/journal.pone.0077351

**Published:** 2013-10-03

**Authors:** Lydia Ferguson, Javier J. How, Alexander I. Agoulnik

**Affiliations:** 1 Department of Human and Molecular Genetics, Herbert Wertheim College of Medicine, Florida International University, Miami, Florida, United States of America; 2 Department of Obstetrics and Gynecology, Baylor College of Medicine, Houston, Texas, United States of America; University of Nevada School of Medicine, United States of America

## Abstract

The environmental niche of the spermatogonial stem cell pool is critical to ensure the continued generation of the germ cell population. To study the consequences of an aberrant testicular environment in cryptorchidism we used a mouse model with a deletion of *Rxfp2* gene resulting in a high intra-abdominal testicular position. Mutant males were infertile with the gross morphology of the cryptorchid testis progressively deteriorating with age. Few spermatogonia were identifiable in 12 month old cryptorchid testes. Gene expression analysis showed no difference between mutant and control testes at postnatal day 10. In three month old males a decrease in expression of spermatogonial stem cell (SSC) markers *Id4*, *Nanos2*, *and Ret* was shown. The direct counting of ID4+ cells supported a significant decrease of SSCs. In contrast, the expression of *Plzf*, a marker for undifferentiated and differentiating spermatogonia was not reduced, and the number of PLZF+ cells in the cryptorchid testis was higher in three month old testes, but equal to control in six month old mutants. The PLZF+ cells did not show a higher rate of apoptosis in cryptorchid testis. The expression of the Sertoli cell *FGF2* gene required for SSC maintenance was significantly reduced in mutant testis. Based on these findings we propose that the deregulation of somatic and germ cell genes in the cryptorchid testis, directs the SSCs towards the differentiation pathway. This leads to a depletion of the SSC pool and an increase in the number of PLZF+ spermatogonial cells, which too, eventually decreases with the exhaustion of the stem cell pool. Such a dynamic suggests that an early correction of cryptorchidism is critical for the retention of the SSC pool.

## Introduction

Cryptorchidism (undescended testes) is the most common congenital abnormality and affects around 2-4% of newborn boys worldwide. If left untreated, cryptorchidism can lead to an increased risk of infertility and testicular cancer [1,2]. The degree of abnormalities strongly correlates with the testicular position of non-scrotal gonads, with testes located in a high intra-abdominal position most affected.

Spermatogenesis in the cryptorchid testis is severely impaired [2,3]. It is generally accepted that the high temperature environment of an intra-abdominal testicular position inhibits the correct maturation and differentiation of germ cells. The most sensitive cells are primary spermatocytes and round spermatids which show early DNA damage after heat stress [4-6], while the spermatogonia and elongated spermatids are more resistant to high temperatures [7]. Many cell signaling pathways critical for spermatogenesis are vulnerable to heat stress and their disruption has been linked to spermatogenic arrest in the cryptorchid testis. The aberrant testicular environment has been shown to have a detrimental effect on testicular somatic cells such as Sertoli and Leydig cell function, that may lead to an inability to support germ cell maintenance and differentiation [1,4].

About half of all cryptorchidism cases in humans naturally resolve within the first year after birth and in many other cases, surgical intervention (orchiopexy) corrects the problem. However when orchiopexy is performed late, some patients present sub- or fully infertile phenotypes [8]. This may be a result of more complex defects in hormonal control or complications from the orchiopexy but may also indicate irreversible damage to the establishing SSC pool during the early neonatal period [2,9].

SSCs make up a small fraction of the total germ cell population and in rodents are attributed to the A_single_ or A_s_ population (A_dark_ in primates). These cells represent around 1 in every 3000 cells in the mouse testis and are the most primitive spermatogonia cells [10]. SSCs are located on the basal membrane within the seminiferous tubules and A_s_-A_al_ (A_aligned_) spermatogonia have been found to be positioned close to the blood vessels and interstitial cells of the testis, possibly to obtain sufficient levels of SSC maintenance factors produced in Sertoli cells [11]. A_s_ cells are capable of either self-renewal or differentiation into two A_paired_ spermatogonia that do not complete cytokinesis and remain conjoined by an intercellular bridge. It is at this point that the spermatogonia are “committed” to differentiation and although the umbrella term “undifferentiated spermatogonia” is often used for all A_s_-A_al_ cells, only A_s_ spermatogonia appear to be the pluripotent stem cells under normal conditions. Some recent evidence also suggests that A_pr_ and potentially A_al_ spermatogonia are able to fragment to generate different populations of As spermatogonia but whether this routinely occurs needs further confirmation [12]. A number of markers expressed in most stages of undifferentiated spermatogonia such as Plzf, Neurog3, Nanos2, Oct4, Ret and Gfrα1 contribute to the identification of the SSC population. Recently, a marker for A_s_ spermatogonia was reported, ID4 - Inhibitor of DNA Binding 4, that identified single stem cells in seminiferous tubule whole-mount immunofluorescence [13]. This is the only known marker believed to be solely expressed in A_s_ stem cells.

The careful balance between SSC self-renewal and the switch to differentiation is influenced by factors comprising the SSC somatic cell niche [14]. It has been shown that GDNF - Glial cell-Derived Neurotrophic Factor, secreted by Sertoli cells, interacts with GFRα1 and RET receptors on undifferentiating spermatogonia and this signaling pathway is essential to promote self-renewal of SSCs [15-18]. Some members of FGF, fibroblast growth factor family such as FGF2, expressed by Sertoli cells, have been shown to play a role in SSC maintenance [19]. CSF1 - Colony-Stimulating Factor 1, secreted by Leydig cells and its receptor CSF1R located on undifferentiated spermatogonia have been also implicated in the proliferation of spermatogonia [20].

We have used cryptorchid mice with a deletion of relaxin family receptor 2 gene, *Rxfp2*, exhibiting high intra-abdominal testes, to analyze changes in both the germ and somatic cells *in vivo* as a result of a non-scrotal testicular environment. We have shown that the number of ID4-positive (ID4+) cells is dramatically reduced with age along with reduced *Id4*, *Nanos3*, and *Ret* gene expression. We did not detect however an increase in the apoptosis of PLZF+ cells. We also demonstrated a reduction in somatic cell growth factor *Fgf2* gene expression in the cryptorchid testis that may alter the balance of SSC self-renewal and differentiation. Thus, the pattern of genes’ expression, involved in SSC self-renewal, suggest that in the cryptorchid testis there is a shift of stem cells towards differentiation leading to a dramatic reduction of SSC pool in cryptorchid testis.

## Materials and Methods

### Ethics Statement

This study was carried out in strict accordance with the recommendations in the Guide for the Care and Use of Laboratory Animals of the National Institutes of Health. The protocol was approved by the Institutional Animal Care and Use Committee of Florida International University (IACUC Protocol Numbers: 09-003 and 12-006).

### Mouse Strains and Breeding

The Crsp mutants have a deletion in mouse chromosome 13 that completely removes the *Rxfp2* gene [21]. The *crsp/crsp* homozygous females were bred with *crsp*/+ heterozygous males to produce homozygotes and heterozygotes. Homozygous males, identified by a white spot were used as an experimental cryptorchid group; the heterozygous male siblings with wild-type phenotype were the controls. The mice were maintained under standard conditions at FIU animal facilities.

### Histology and Immunohistochemistry

Testes were isolated and fixed overnight in Bouin’s solution, then washed 3 times in PBS, and stored overnight in 70% ethanol. The tissues were processed and embedded in paraffin before being sectioned at 7 µM and fixed to a slide. Slide sections were deparaffinized, rehydrated in graded ethanol, and stained with Haematoxylin and Eosin. Then sections were dehydrated in ethanol and Histoclear before adding mounting serum and a coverslip.

Immunohistochemistry (IHC) was performed using PLZF and ID4 antibody (Santa Cruz Biotechnology, Santa Cruz, CA). Vectastain ABC (avidin–biotin–peroxidase) kit (Vector Laboratories, Burlingame, CA) was used for detection and used as per manufacturers guidelines. The color was developed with diaminobenzidine (DAB) as chromogen and sections were then counterstained with Harris Hematoxylin. Analysis was performed on 3 heterozygous control males and 3 homozygous mutant males at day 10, and 4 heterozygous control males and 3 homozygous mutant males at 6 months and 2 each of homozygous mutants and heterozygous control males at 1 year. At least 200 round cross sections of seminiferous tubules were counted per individual male. A Carl Zeiss Axio A1 Microscope and an AxioCam MRc5 CCD camera were used to examine the sections.

### RNA Isolation and quantitative RT-PCR

Extracted testis was homogenized in 1ml Trizol reagent (Invitrogen, Carlsbad, CA) for approximately 30 s until no tissue clumps were visible. Chloroform (100 µl) was added and the sample was vigorously shaken for 15 s, incubated for 3 min at room temperature and centrifuged at 12,000 rpm for 15 min at 4 °C. The top aqueous layer containing the RNA was removed and added to an equal volume of isopropanol, mixed by inversion and incubated at room temperature for 10 min. Samples were then transferred to an RNeasy spin column (Qiagen RNeasy Kit, Quagen, Valencia, CA) and centrifuged for 15 s at 10,000 rpm before resuming the Qiagen RNeasy kit protocol. Samples were then treated with *DNaseI* (Fermentas, Pittsburgh, PA) and cDNA was synthesized using Verso cDNA kit (Thermo Scientific, Waltham, MA).

Quantitative PCR was performed using GoTaq qPCR Master Mix (Promega, Madison, WI), and gene specific primers in a RealPlex^2^ Mastercycler (Eppendorf, Westbury, NY) according to the manufacturer’s instructions. The sequences of the PCR primers are shown in Table 1. Three dilutions of RNA were used as standards and values were normalized to *β-actin, Plzf*, or *clusterin* gene expression. Results were analyzed using GraphPad Prism software (GraphPad Software, La Jolla, CA) for statistical significance using Student’s *t* test.

**Table 1:** PCR primers used analysis of gene expression.

**Gene**	**Forward**	**Reverse**
*Gfrα1*	ACTCCTGGATTTGCTGATGTCGG	CGCTGCGGCACTCATCCTT
*Fgf9*	TGCAGGACTGGATTTCATTTAG	TGCAGGACTGGATTTCATTTAG
*Ret*	TCCCTTCCACATGGATTGA	ATCGGCTCTCGTGAGTGGTA
*Fgf2*	CGGCTCTACTGCAAGAACG	TGCTTGGAGTTGTAGTTTGACA
*Csf1*	CAACAGCTTTGCTAAGTGCTCTA	CACTGCTAGGGGTGGCTTTA
*Nanos2*	GACCATCCATCTATCTTCACCT	CCTCCTCTAGTTCCTGTAACC
*Neurog3*	GCTATCCACTGCTGCTTGA	CCGGGAAAAGGTTGTTGTGT
*Gdnf*	TCCAACTGGGGGTCTACG	GACATCCCATAACTTCATCTTAGAGTC
*Fgfr2*	CCTGCGGAGACAGGTAACA	CGGGGTGTTGGAGTTCAT
*Id4*	AGGGTGACAGCATTCTCTGC	CCGGTGGCTTGTTTCTCTTA
*Plzf*	GAGACACACAGACAGACCCATACT	CACACATAACACAGGTAGAGGTACG
*Clusterin*	CGCTATAAATAGGGCGCTTC	GCCTCCTTGGAATCTGGAGT

### Flow Cytometry

The testes were decapsulated and placed in 3 ml Gey’s balanced salt solution (GBSS, Sigma-Aldrich, St. Louis, MO) containing 1.0 mg/ml Collagenase type IV (Sigma) and 10 µl DNase I (10 µg/µl) and agitated for 15 min at 32°C. The tube was then allowed to stand for 5 minutes and the supernatant discarded. Tubules were washed twice in 1x PBS and then resuspended in 3 ml PBS, 5 µl trypsin (50 mg/ml), 10 µl DNAse I and 1.5 µl 2M HCl and incubated for 15 min at 32°C. 400µl fetal calf serum (FCS, Hyclone, Thermo Scientific) was then added to the suspension to stop trypsin activity and mixed by gentle pipetting. The cell suspension was filtered through 100 µm nylon mesh to remove large clumps of cells and then washed twice with PBS by 1000 rpm at room temperature for 4 min. Cells were counted in the second wash, prior to spinning. Cells were resuspended in Cytofix/Cytoperm solution (BD Biosciences, San Jose, CA) at 2 x 10^6^ cells/ml and incubated on ice for 20 min. Cells were pelleted and Cytofix/Cytoperm solution was discarded before the cells were washed twice at room temperature with 0.5 ml BD Perm/Wash buffer per 1 x 10^6^ cells and supernatants discarded. BD Perm/Wash buffer was used to resuspend the cells at 5 x 10^6^ cells/ml and samples were aliquot at 500,000 cells per 100 µl test. Samples were treated for 30 min with either 5 µl Alexa Fluor 647 Mouse Anti-Cleaved PARP (Asp 214) (BD Pharmingen, Franklin Lakes, NJ), 5 µl PLZF rabbit polyclonal antibody (Santa Cruz) with 0.5 µl Alexa Fluor 488 goat anti-rabbit IgG (Invitrogen) or 5 µl Propidium Iodide staining solution (50 µg/ml, BD BioSciences), or a sequence of all three. Data (100,000 events) were collected on a BD Accuri C6 flow cytometer (BD Biosciences) and analyzed using CFlow Plus software.

## Results

### Abnormal spermatogenesis in Crsp cryptorchid males

Mice with a Crsp mutation contain an insertion of tyrosinase minigene in the proximal part of chromosome 5. This integration is concomitant with a 550 kb deletion that completely removes the relaxin family G protein-coupled receptor for INSL3 hormone [21-23]. Male homozygotes, recognized by white spotting, fail to undergo normal testicular descent and are cryptorchid. These mice are infertile and their testes are located in a high intra-abdominal position. The heterozygous males have a normal scrotal testis position and are fully fertile.

**Figure 1:**
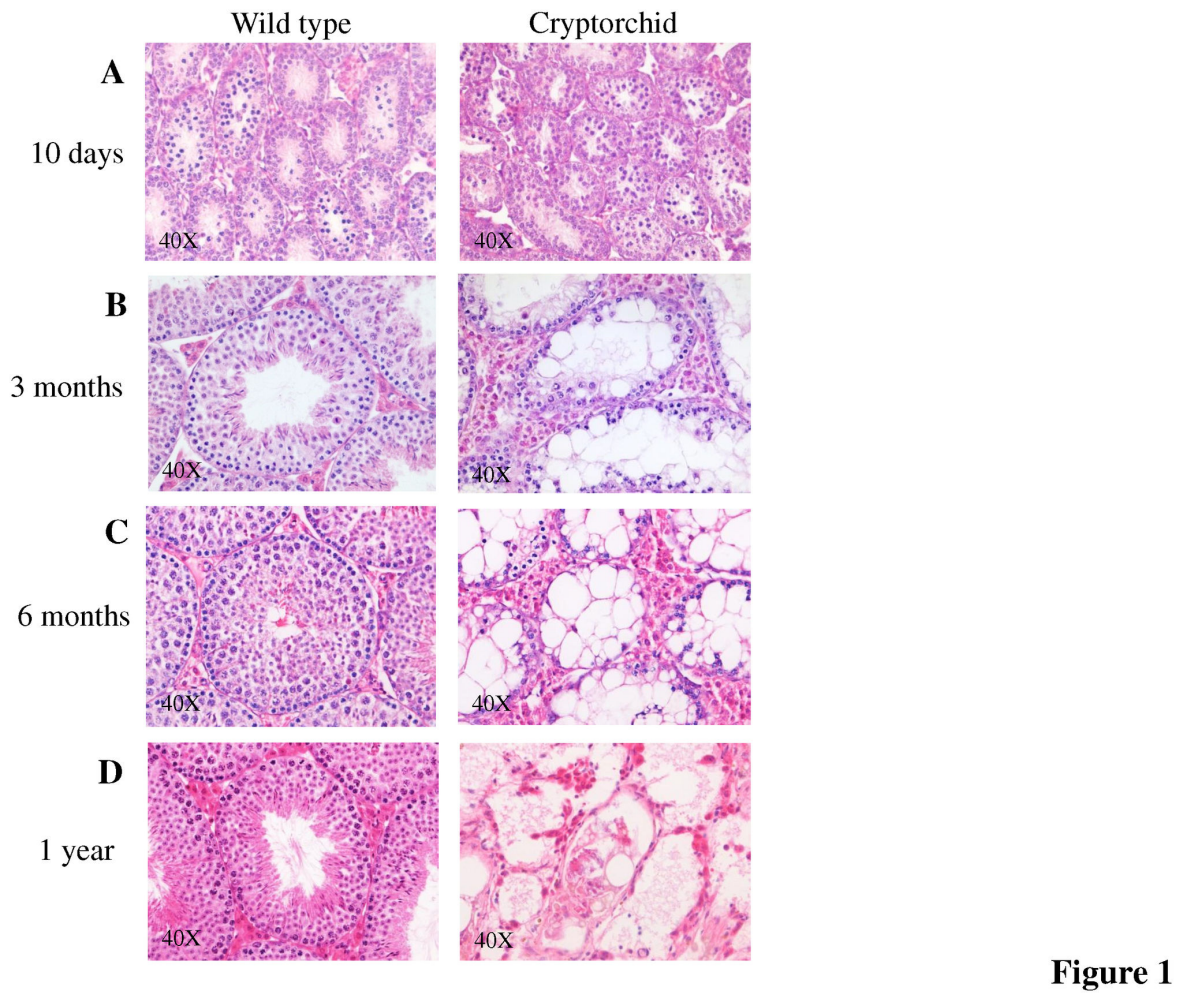
Progressive degeneration of cryptorchid testis. (**A**) Male cryptorchid testis shows no differences at day 10 compared to wild-type control. (**B**) By 3 months fewer differentiating germ cells are present in cryptorchid testes with big Sertoli cell intracellular vacuoles and dilated intercellular spaces. The germ cells are almost all absent by 6 months (**C**). (**D**) One year old cryptorchid testis showed severely disrupted tubular structure with single germ cells.

Histologically, at day 10, the mutant males showed no abnormalities in their testes compared to age matched control males (Figure 1) and the testes were of similar weight and appearance. The testes isolated from the cryptorchid adult (3 and 6 months old and 1 year) males were smaller in size and weighed four times less at 6 months (N=3 in each group P<0.0001) than the control sibling testes and at one year old, the testes of the mutants weighed ten times less than those of the control sibling testes. Histological analysis of adult mutant testes revealed a severely abnormal phenotype (Figure 1). The Sertoli cells were highly vacuolated with a significant increase in number of Leydig cells. At 3 months some of the tubules contained what appeared to be differentiating spermatogonia and spermatocytes. At 6 months the seminiferous tubules were almost devoid of any differentiating germ cells and the tubules lacked structure and uniformity. Few spermatogonial cells with dark stained nuclei were located close to the basement membrane. By 1 year, it was difficult to identify individual seminiferous tubules and very few germ cells were identified in the tubules that were present. Detached germ cells not enclosed within seminiferous tubules were also visible and much cell debris was present.

The timepoints chosen for analysis reflect distinctive stages of testis development. At 10 days after birth the process of testicular descent in mice is fully completed [24,25]. The testes are located in the scrotum and the gonocytes are differentiated into spermatogonial cells including a primary pool of SSCs. The second stage of 3 months reflects the full spectrum of abnormalities usually observed in adult cryptorchid testis. Significantly, at this stage the cryptorchid mice retain a significant number of spermatogonial cells. The third and fourth time points of 6 months and 1 year respectively, identifies abnormalities observed in the mature and then ageing cryptorchid testis.

### Cryptorchidism causes deregulation of gene expression

**Figure 2:**
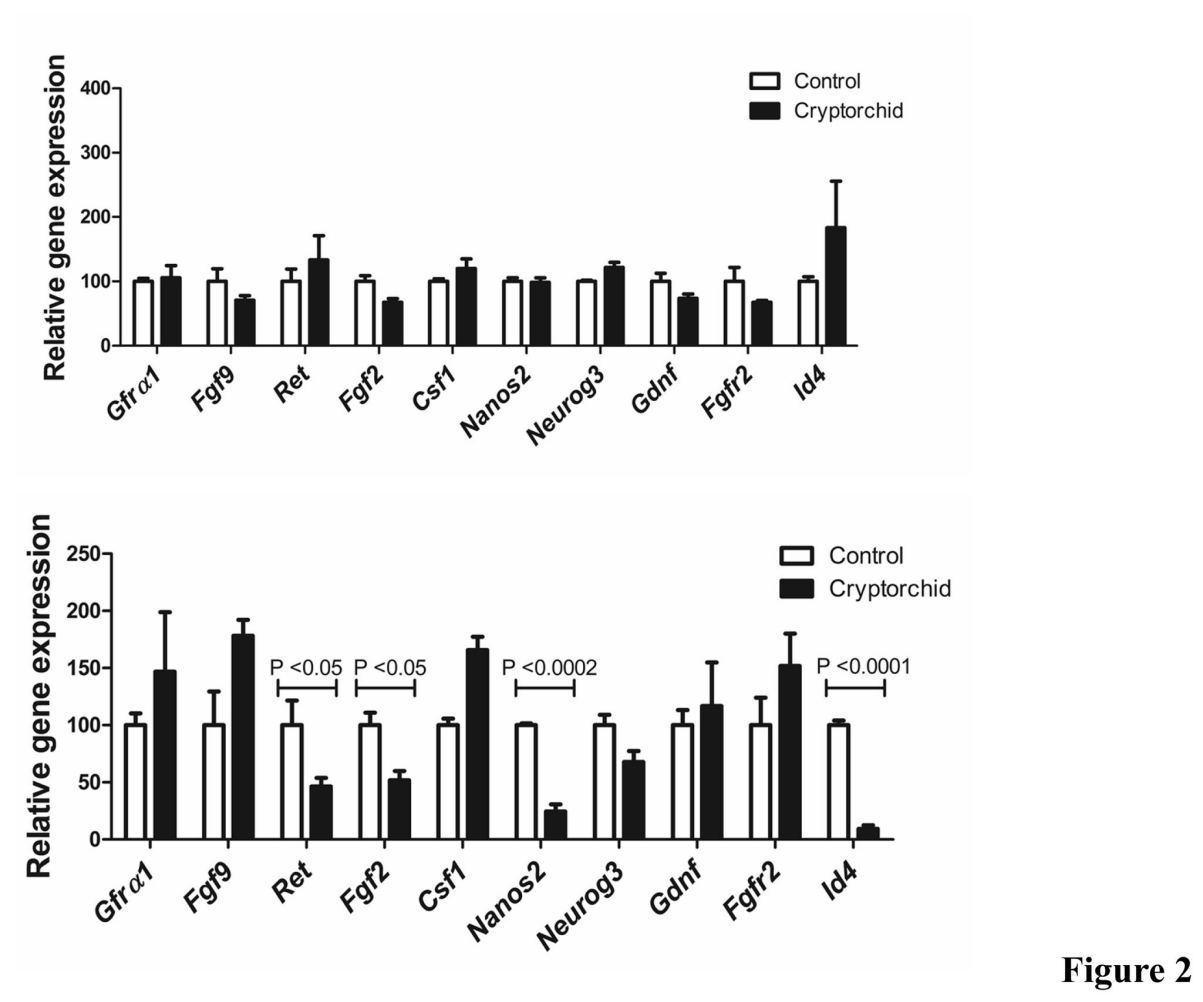
Analysis of testicular germ and somatic gene expression in cryptorchid testes. Real time qRT-PCR analysis of spermatogonial stem cell markers and growth factors for 10 days old (**A**) and 6 month old cryptorchid and control sibling mice (**B**). Data represent the mean ± SEM.

We performed a series of quantitative PCR experiments on whole testis RNA from day 10 and 3 months old adult cryptorchid and control males (Figure 2). Genes known to contribute to a role in spermatogonial stem cell maintenance were selected for investigation. The expression of the germ cell-specific markers was normalized to the *Plzf* gene, the expression of the Sertoli markers to *clusterin.*

First, we tested the expression of spermatogonia specific markers. *Id4* is a recently identified stem cell marker with expression restricted to A_s_ spermatogonia and *Nanos2* expression has been shown to be restricted to a primitive subset of undifferentiated spermatogonia. Both *Id4* and *Nanos2* were significantly down-regulated in adult males compared to the control males (P<0.0001 and P<0.0002 respectively) whereas in day 10 mutant males neither gene was significantly altered. A decrease in the expression of both markers in mutant testes suggested a reduction of A_s_ spermatogonial stem cells in the cryptorchid males compared with their age matched sibling controls.

Glial cell line-derived neurotrophic factor (*Gdnf*) is expressed in Sertoli cells and is thought to be crucial for the maintenance and self-renewal of the stem cell population. GDNF interacts with *Gdnf* family receptor α1 (*GFRα1*) and the co-receptor *Ret*, both shown to be expressed in undifferentiated SSCs. Neither *Gdnf* nor *Gfrα1* expression was significantly altered in the adult mutant males, however *Ret* expression was significantly down-regulated compared to wild-type controls.

An additional marker for undifferentiated SSCs *Neurog3*, had only slightly reduced expression in cryptorchid males. This marker is associated with later stage SSCs committed to differentiation and was less affected by the cryptorchid phenotype than *Id4* and *Nanos2*, indicating that the most dramatic changes were in the primitive stem cell populations.

Members of the *Fgf* family expressed in different somatic cells of the testis were also analyzed. *Fgf2* was expressed at a lower level in the adult cryptorchid males when normalized to the Sertoli cell gene, *clusterin* (P=0.0297).

### Changes in spermatogonial cells in cryptorchid testis

**Figure 3:**
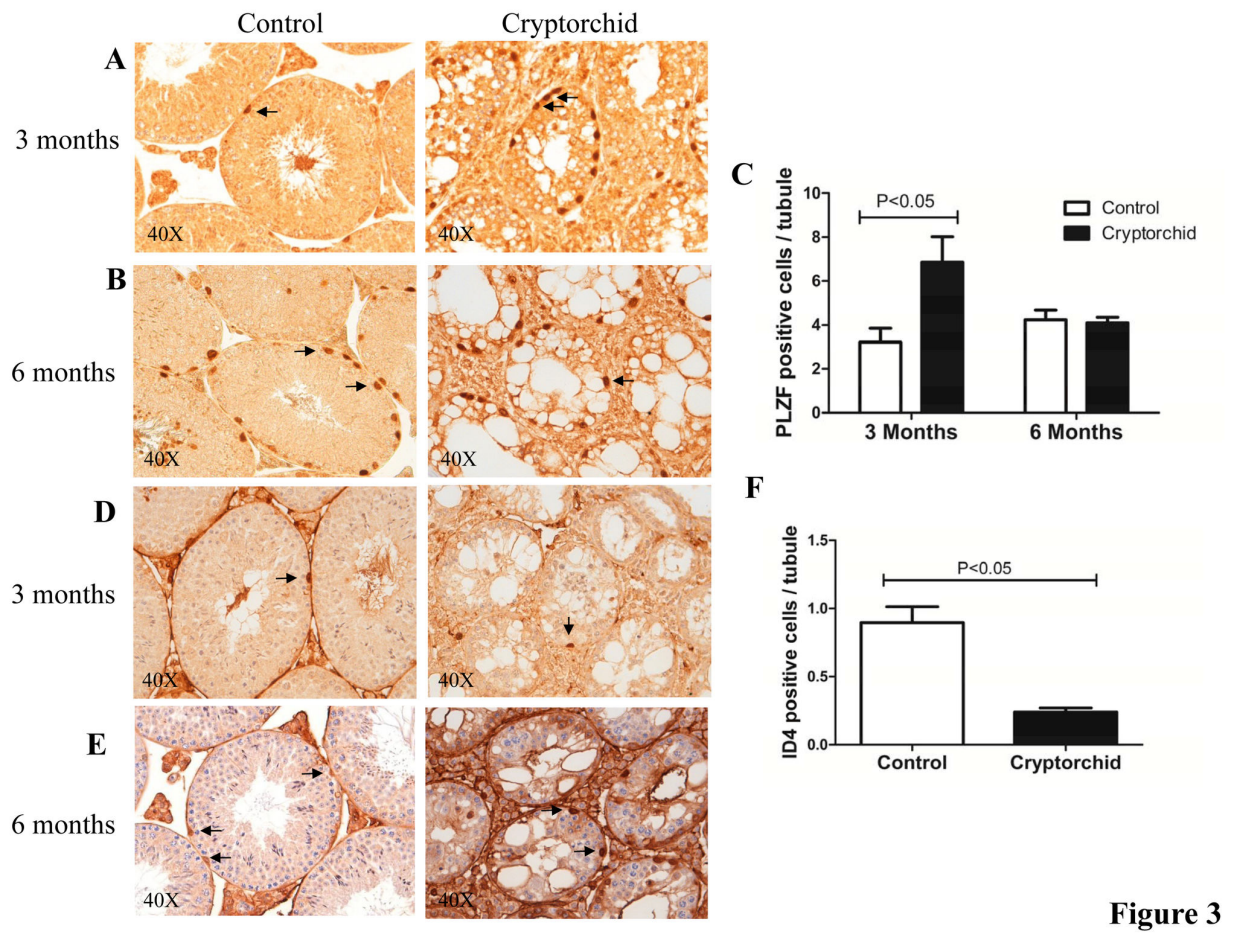
Immunohistochemistry (IHC) analysis for PLZF and ID4 staining in cryptorchid testes. Cross sections of cryptorchid and control testes stained with antibody for PLZF at 3 months (**A**) and at 6 months (**B**). Quantitative comparison of PLZF positive cells (arrows) per seminiferous tubule between cryptorchid and control mice at 3 months and 6 months (**C**). Cross sections of cryptorchid and control testes stained with antibody for ID4 at 3 months (**D**) and at 6 months (**E**). Quantitative comparison of ID4 positive cells (arrows) per tubule between cryptorchid and control mice at 3 months and 6 months (**F**). Data represent the mean ± SEM.

We used immunohistochemistry to determine if the numbers of cells positive for the spermatogonial A_s_-A_al_ marker PLZF were altered in cryptorchid testes. While day 10 males showed no change in the number of positive cells, the 3 month old males had significantly more PLZF+ cells per tubule compared to age matched controls (Figure 3A). Cells positive for PLZF were positioned at the basal membrane of the seminiferous tubules and were easy to identify in the adult male controls. In cryptorchid testis some PLZF+ cells had detached from the basement membrane and were aberrantly positioned in the middle of the tubules. Interestingly, mutant males aged to 6 months showed no significant change in PLZF+ cells compared to age matched sibling controls.

Cryptorchid adult mutant males had a significant reduction in the mRNA level of the A_s_ spermatogonial marker ID4, indicating either a reduction in gene expression, or fewer ID4+ cells in the mutant males. The reduced number of ID4 positive cells per tubule at 3 months, confirmed that SSC pool was significantly diminished (P = 0.0053) (Figure 3B). At 6 months, the level of background staining and the weak staining for ID4 in the cryptorchid sections hampered an accurate quantitation of ID4+ cells.

### PLZF+ cells undergo same rate of apoptosis in cryptorchid and normal testis

**Figure 4:**
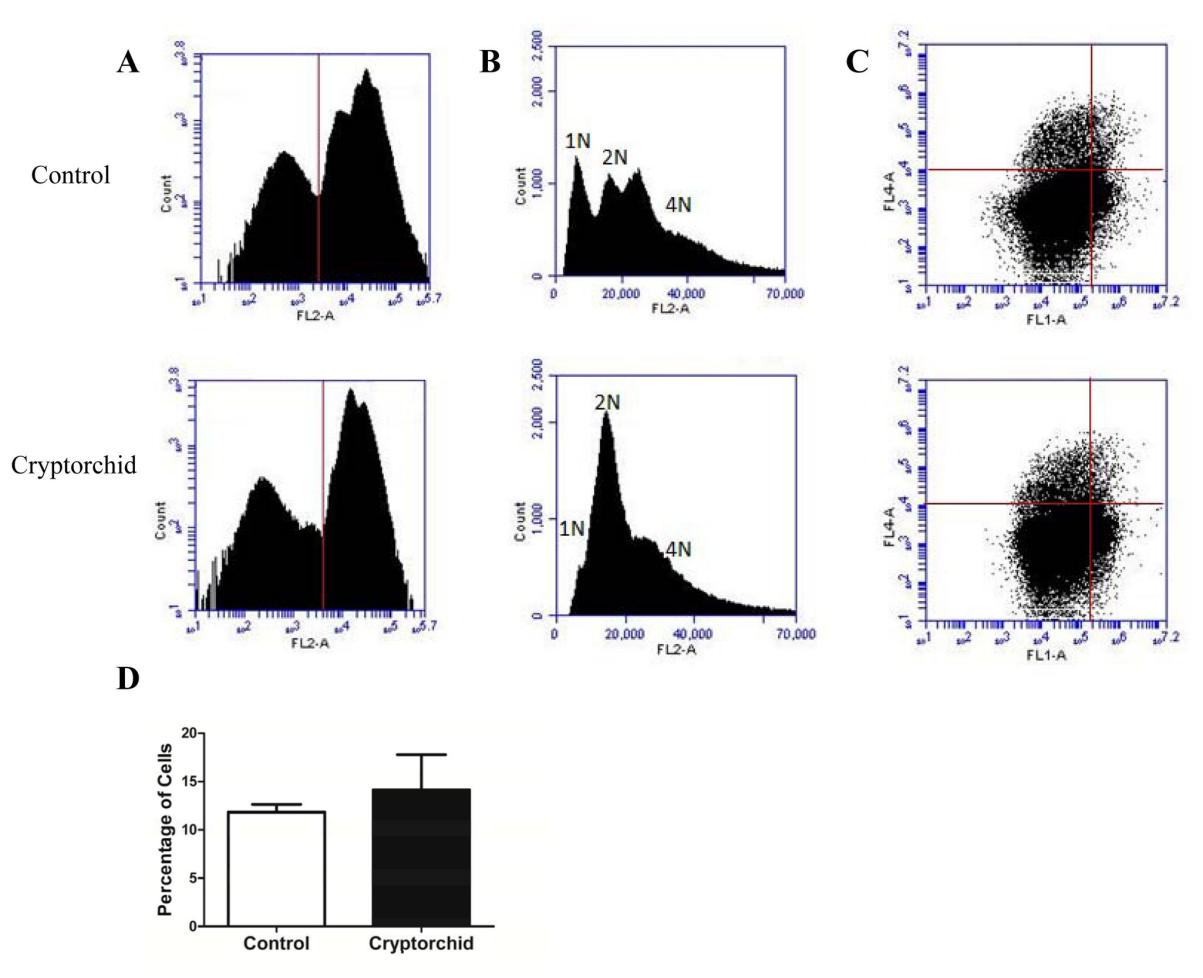
No increase of apoptosis in spermatogonial cells in cryptorchid testes. Flow cytometry analysis of control and cryptorchid whole testes from adult mice using propidium iodide (PI) and PLZF and cleaved PARP antibodies. (**A**) Flow cytometric histogram showing cells positive for PI (right of the vertical line) and selected for further analysis for PLZF and cleaved PARP. (**B**) Representative flow cytometric histogram of cells positive for PI showing a distribution for 1N, 2N and 4N cells in the control testes and a higher number of 2N cells and fewer 1N cells in the cryptorchid testes. (**C**) Dual parameter flow cytometric analysis of PI positive cells to select cells positive for PLZF (fl1) and cleaved PARP (fl4). Red lines indicate gating boundaries for each fluorescence channel. (**D**) The rate of apoptosis (cleaved PARP+ cells) in PLZF+ cells is the same in control and cryptorchid testes. Data represent the mean ± SEM of testicular cell pools from three month old control (N=3) and cryptorchid (N=3) males.

To determine whether PLZF+ cells were undergoing apoptosis in the testis of the cryptorchid mice at the same rate as those in the testis of age matched control mice, we performed flow cytometry combining markers for PLZF, PARP (Asp 214) and Propidium Iodide (PI). Control markers were each used individually on each sample, to determine the optimal settings for each marker. Cells were sorted according to the intensity of the DNA stain PI, and events that displayed a low intensity were excluded from the population to ensure only intact, cells were included in the analysis (Figure 4A). Histograms of the PI populations showed an even distribution of haploid (1N), diploid (2N) and tetraploid (4N) nuclear DNA content in cells from the control mice, whereas the mutant mice showed a dramatic depletion of haploid cells and an increase in the relative number of diploid cells (Figure 4B). The population of PI positive cells was then used to determine the percentage of PLZF positive cells that were positive for the pro-apoptotic marker – PARP using dual parameter flow cytometric analysis. Cells that were clustered in the upper right quadrant that had the highest intensity for each marker were classified as positive for PLZF and PARP (Figure 4C). We found that the number of PLZF positive cells undergoing apoptosis in the cryptorchid testes (14.1%) was not significantly different from the normal testes (11.8%) (Figure 4D).

## Discussion

It has been shown that early surgical correction of undescended testis in affected boys has a beneficial effect on their adult fertility. It has been proposed that the depletion of the SSC pool may be responsible for declining fertility in the cryptorchid gonads [2,9]. We have investigated the effect of cryptorchidism on the development of SSCs in the testis in males homozygous for the Crsp mutation. Such animals have a complete deletion of the *Rxfp2* gene, the receptor for INSL3 hormone controlling gubernacular differentiation and testicular descent [22]. Comparison of cryptorchid and wild-type testes showed profound changes in testicular structure with the severity of abnormalities increasing with the age of the animals. Gene expression analysis revealed changes in genes expressed by and known to regulate SSCs self-renewal. The decrease in the earliest spermatogonial markers’ expression correlated with the dramatic reduction of A_s_ spermatogonial cell number. Additionally, we did not detect an increase in apoptosis rates in differentiating spermatogonia in young adult males, suggesting that the reduction of spermatogonial cells in cryptorchid testis is due to the depletion of SSC pool caused by the shift from their renewal to differentiation. These differentiating spermatogonia then accumulate in the cryptorchid testis before eventually undergoing apoptosis.

The identification of the true germ stem cell population in mammalian testis has been somewhat elusive due to a posited shift in SSC gene expression as opposed to a definitive stem cell marker. Importantly, three markers, down-regulated in the cryptorchid testis in our study not only define the SSC population, but also have an important functional role in their renewal.

Oatley et al recently demonstrated that the inhibitor of DNA 4 (ID4) expression is specific to A_s_ spermatogonia in the testis [13]. Importantly, ID4+ cells were also PLZF+. In our study, the expression of this marker was dramatically reduced at the RNA level in the cryptorchid testis. In parallel, the number of ID4+ cells defined by IHC was also dramatically reduced. Previously, an Id4^-/-^ knockout mouse showed decreasing fertility with age with only 25% of aged mice retaining fertility compared to 100% of heterozygous controls. Interestingly, the Id4^-/-^ males exhibit a subfertile phenotype but are not fully infertile [13]. The number of PLZF+ cells in 2-3 month old Id4^-/-^ males was significantly higher than that in the *Id4* ^*+/-*^ heterozygous control males. As the mice aged, they then saw a decrease in the number of PLZF+ cells in the mutants compared to the controls [13]. In our experiments we discovered the same dynamics: an initial increase in PLZF+ cells in cryptorchid males at 3 months of age, followed by their gradual decrease to almost equal to control numbers at 6 months. At 12 months only rare germ cells can be found in mutant testis. Given that the expression of *Id4* was not completely suppressed in our model, it may require more time to see a complete absence of SSCs.

Another marker affected by cryptorchidism is NANOS2, an RNA binding protein. It is expressed predominantly in A_s_ and A_pr_ spermatogonia and is also suggested to play an important role in maintaining stem cells in their quiescent state [26]. Nanos2^-/-^ male germ cells are lost at the beginning of embryonic day 15.5 (E15.5) and are completely depleted by birth [27]. Postnatal conditional ablation of *Nanos2* depleted SSC reserves, whereas *Nanos2* overexpression resulted in the accumulation of spermatogonia with stem cell-like properties [26]. Based on this data, we can hypothesize that in cryptorchid males, the decrease in NANOS2 expression may contribute to the induction of SSC differentiation. NANOS2 is regulated by and acts downstream of GDNF signaling [28]. Importantly, in cryptorchid testis the expression of RET, one of the GDNF co-receptors was also downregulated.

The significance of the testicular somatic cell niche in stem germ cell maintenance is well- recognized. The selection between self-renewal and differentiation is regulated by a growth factor milieu supplied by different somatic cells [29]. Specifically, Sertoli cells are believed to be a key support cell population not only secreting soluble factors but also coordinating the signaling of other somatic cells. Among them are GDNF and FGF2, the most studied regulators of SSC self-renewal [19,30]. The different cellular composition of cryptorchid and control testes makes it difficult, however, to accurately assess the changes in gene expression of various cell-specific markers when using total testicular RNA samples. The cryptorchid testis did not contain any haploid germ cells and the proportion of interstitial Leydig cells was significantly increased. Taking this into consideration we normalized the Sertoli cell gene expression using the cell-specific marker *clusterin*. While the expression of *Gdnf* did not change, the expression of *Fgf2* was significantly reduced in adult cryptorchid Sertoli cells. Thus, decreased FGF2 production coupled with reduced GDNF signaling due to lower expression of RET may explain a failure to re-capitulate the SSC population in the cryptorchid testis.

It is generally assumed that the high temperature environment of the cryptorchid testis causes an activation of apoptotic pathway in germ cells. Such scenario would ultimately also lead to the depletion of SSCs after their differentiation. To analyze the contribution of cell apoptosis in SSC fate we used flow cytometry to calculate the proportion of double labeled testicular spermatogonial PLZF+, pro-apoptotic cleaved PARP+ cells among PLZF+ cells. We found no difference in these ratios in cryptorchid and scrotal testes.

Our results appear to contradict the well-described phenomenon of increased SSC number in mice with surgically induced cryptorchidism [31,32]. There are however several critical differences between these two models. The surgery in the wild-type males was performed in sexually mature 6-8 months old animals, whereas in Crsp mice the cryptorchidism is inborn and thus the SSC population in non-descended testes is subjected to an abnormal environment from birth. The spermatogonial cell analysis was performed in induced cryptorchid mice after another 2-3 months. In the Crsp model in 3 months old cryptorchid males we also saw an increase of PLZF+ spermatogonial cells, the same phenomenon observed in the experimental cryptorchidism 2-3 months after surgery. One can speculate that similar processes are taking place in induced cryptorchidism – a gradual shift of SSC to differentiation, depletion of the A_s_ pool and an increase of A_pr_ and A_al_ cells which can contribute to stem cell population [12] in transplantation experiments.

The other important question is whether the findings described here are specific for RXFP2-deficient mice or if they truly reflect the fate of SSCs in any cryptorchid testis. We have demonstrated that conditional gene targeting of *Rxfp2* in germ cells has no direct effect on spermatogenesis itself in scrotal testes [33]. Moreover, the analysis of *Rxfp2* gene expression using knock-in LacZ reporter failed to detect expression of the gene in spermatogonial or Sertoli cells [33]. It is unlikely, therefore, that the INSL3-RXFP2 signaling plays any role in SSC pool maintenance. Recently, similar to our data, in rats, where cryptorchidism was induced by flutamide injections into pregnant females, a decrease of undifferentiated embryonic cell transcription factor 1 (UTF1) positive early spermatogonia cells was detected [9]. Further analysis of surgically corrected testes at different ages in our and other cryptorchid animal models as well as in experimentally induced cryptorchidism might be needed to understand the full effect of abnormal testis position on SSC fate.

In summary, we suggest that the abnormal environment of the cryptorchid testis leads to a deregulation of a number of genes expressed in both germ and somatic cells involved in the maintenance of the SSC pool. This in turn directs the SSCs towards the differentiation pathway, leading to a depletion of ID4 positive A_s_ stem cells and an initial increase in PLZF+ differentiating spermatogonia. The total spermatogonial cell number decreases with age, as the SSC pool is reduced. Such events might explain the importance of early intervention and surgical correction of cryptorchidism in human patients.

## References

[1] Ferguson L, Agoulnik AI (2013) Testicular cancer and cryptorchidism. Front Endocrinol (Lausanne)4: 32. 10.3389/fendo.2013.00032 PMC3602796 23519268

[2] Agoulnik AI, Huang Z, Ferguson L (2012) Spermatogenesis in cryptorchidism. Methods Mol Biol 825: 127-147. doi:10.1007/978-1-61779-436-0_11. PubMed: 22144242. 22144242

[3] Nishimune Y, Aizawa S, Komatsu T (1978) Testicular germ cell differentiation in vivo. Fertil Steril 29: 95-102. PubMed: 23321. 23321 10.1016/s0015-0282(16)43045-1

[4] Cataldo L, Mastrangelo MA, Kleene KC (1997) Differential effects of heat shock on translation of normal mRNAs in primary spermatocytes, elongated spermatids, and Sertoli cells in seminiferous tubule culture. Exp Cell Res 231: 206-213. doi:10.1006/excr.1996.3447. PubMed: 9056428. 9056428

[5] Yin Y, Hawkins KL, DeWolf WC, Morgentaler A (1997) Heat stress causes testicular germ cell apoptosis in adult mice. J Androl 18: 159-165. PubMed: 9154510. 9154510

[6] Li Y, Zhou Q, Hively R, Yang L, Small C et al. (2009) Differential gene expression in the testes of different murine strains under normal and hyperthermic conditions. J Androl 30: 325-337. doi:10.1111/j.1439-0272.1998.tb01178.x. PubMed: 19096088. 19096088 PMC3209712

[7] Pérez-Crespo M, Pintado B, Gutiérrez-Adán A (2008) Scrotal heat stress effects on sperm viability, sperm DNA integrity, and the offspring sex ratio in mice. Mol Reprod Dev 75: 40-47. doi:10.1002/mrd.20759. PubMed: 17474098. 17474098

[8] Taran I, Elder JS (2006) Results of orchiopexy for the undescended testis. World J Urol 24: 231-239. doi:10.1007/s00345-006-0056-4. PubMed: 16676187. 16676187

[9] Kamisawa H, Kojima Y, Mizuno K, Imura M, Hayashi Y et al. (2012) Attenuation of spermatogonial stem cell activity in cryptorchid testes. J Urol 187: 1047-1052. doi:10.1016/j.juro.2011.10.170. PubMed: 22266011. 22266011

[10] Tegelenbosch RA, de Rooij DG (1993) A quantitative study of spermatogonial multiplication and stem cell renewal in the C3H/101 F1 hybrid mouse. Mutat Res 290: 193-200. doi:10.1016/0027-5107(93)90159-D. PubMed: 7694110. 7694110

[11] Chiarini-Garcia H, Hornick JR, Griswold MD, Russell LD (2001) Distribution of type A spermatogonia in the mouse is not random. Biol Reprod 65: 1179-1185. doi:10.1095/biolreprod65.4.1179. PubMed: 11566741. 11566741

[12] de Rooij DG, Griswold MD (2012) Questions about spermatogonia posed and answered since 2000. J Androl 33: 1085-1095. doi:10.2164/jandrol.112.016832. PubMed: 22879526. 22879526

[13] Oatley MJ, Kaucher AV, Racicot KE, Oatley JM (2011) Inhibitor of DNA binding 4 is expressed selectively by single spermatogonia in the male germline and regulates the self-renewal of spermatogonial stem cells in mice. Biol Reprod 85: 347-356. doi:10.1095/biolreprod.111.091330. PubMed: 21543770. 21543770 PMC3142260

[14] Oatley MJ, Racicot KE, Oatley JM (2011) Sertoli cells dictate spermatogonial stem cell niches in the mouse testis. Biol Reprod 84: 639-645. doi:10.1095/biolreprod.110.087320. PubMed: 21084712. 21084712 PMC3062034

[15] Grasso M, Fuso A, Dovere L, de Rooij DG, Stefanini M et al. (2012) Distribution of GFRA1-expressing spermatogonia in adult mouse testis. Reproduction 143: 325-332. doi:10.1530/REP-11-0385. PubMed: 22143971. 22143971

[16] Jijiwa M, Kawai K, Fukihara J, Nakamura A, Hasegawa M et al. (2008) GDNF-mediated signaling via RET tyrosine 1062 is essential for maintenance of spermatogonial stem cells. Genes Cells 13: 365-374. doi:10.1111/j.1365-2443.2008.01171.x. PubMed: 18363967. 18363967

[17] Meng X, Lindahl M, Hyvönen ME, Parvinen M, de Rooij DG et al. (2000) Regulation of cell fate decision of undifferentiated spermatogonia by GDNF. Science 287: 1489-1493. doi:10.1126/science.287.5457.1489. PubMed: 10688798. 10688798

[18] Naughton CK, Jain S, Strickland AM, Gupta A, Milbrandt J (2006) Glial cell-line derived neurotrophic factor-mediated RET signaling regulates spermatogonial stem cell fate. Biol Reprod 74: 314-321. doi:10.1095/biolreprod.105.047365. PubMed: 16237148. 16237148

[19] Ishii K, Kanatsu-Shinohara M, Toyokuni S, Shinohara T (2012) FGF2 mediates mouse spermatogonial stem cell self-renewal via upregulation of Etv5 and Bcl6b through MAP2K1 activation. Development 139: 1734-1743. doi:10.1242/dev.076539. PubMed: 22491947. 22491947

[20] Kokkinaki M, Lee TL, He Z, Jiang J, Golestaneh N et al. (2009) The molecular signature of spermatogonial stem/progenitor cells in the 6-day-old mouse testis. Biol Reprod 80: 707-717. doi:10.1095/biolreprod.108.073809. PubMed: 19109221. 19109221 PMC6322428

[21] Overbeek PA, Gorlov IP, Sutherland RW, Houston JB, Harrison WR et al. (2001) A transgenic insertion causing cryptorchidism in mice. Genesis 30: 26-35. doi:10.1002/gene.1029. PubMed: 11353515. 11353515

[22] Gorlov IP, Kamat A, Bogatcheva NV, Jones E, Lamb DJ et al. (2002) Mutations of the GREAT gene cause cryptorchidism. Hum Mol Genet 11: 2309-2318. doi:10.1093/hmg/11.19.2309. PubMed: 12217959. 12217959

[23] Bogatcheva NV, Truong A, Feng S, Engel W, Adham IM et al. (2003) GREAT/LGR8 is the only receptor for insulin-like 3 peptide. Mol Endocrinol 17: 2639-2646. doi:10.1210/me.2003-0096. PubMed: 12933905. 12933905

[24] Kaftanovskaya EM, Feng S, Huang Z, Tan Y, Barbara AM et al. (2011) Suppression of Insulin-like3 receptor reveals the role of beta-catenin and Notch signaling in gubernaculum development. Mol Endocrinol 25: 170-183. doi:10.1210/me.2010-0330. PubMed: 21147849. 21147849 PMC3089031

[25] Kaftanovskaya EM, Huang Z, Barbara AM, De Gendt K, Verhoeven G et al. (2012) Cryptorchidism in mice with an androgen receptor ablation in gubernaculum testis. Mol Endocrinol 26: 598-607. doi:10.1210/me.2011-1283. PubMed: 22322597. 22322597 PMC3327355

[26] Sada A, Suzuki A, Suzuki H, Saga Y (2009) The RNA-binding protein NANOS2 is required to maintain murine spermatogonial stem cells. Science 325: 1394-1398. doi:10.1126/science.1172645. PubMed: 19745153. 19745153

[27] Tsuda M, Sasaoka Y, Kiso M, Abe K, Haraguchi S et al. (2003) Conserved role of nanos proteins in germ cell development. Science 301: 1239-1241. doi:10.1126/science.1085222. PubMed: 12947200. 12947200

[28] Sada A, Hasegawa K, Pin PH, Saga Y (2012) NANOS2 acts downstream of glial cell line-derived neurotrophic factor signaling to suppress differentiation of spermatogonial stem cells. Stem Cells 30: 280-291. doi:10.1002/stem.790. PubMed: 22102605. 22102605

[29] Oatley JM, Brinster RL (2012) The germline stem cell niche unit in mammalian testes. Physiol Rev 92: 577-595. doi:10.1152/physrev.00025.2011. PubMed: 22535892. 22535892 PMC3970841

[30] Kubota H, Avarbock MR, Brinster RL (2004) Growth factors essential for self-renewal and expansion of mouse spermatogonial stem cells. Proc Natl Acad Sci U S A 101: 16489-16494. doi:10.1073/pnas.0407063101. PubMed: 15520394. 15520394 PMC534530

[31] Shinohara T, Avarbock MR, Brinster RL (2000) Functional analysis of spermatogonial stem cells in Steel and cryptorchid infertile mouse models. Dev Biol 220: 401-411. doi:10.1006/dbio.2000.9655. PubMed: 10753526. 10753526

[32] Orwig KE, Ryu BY, Master SR, Phillips BT, Mack M et al. (2008) Genes involved in post-transcriptional regulation are overrepresented in stem/progenitor spermatogonia of cryptorchid mouse testes. Stem Cells 26: 927-938. doi:10.1634/stemcells.2007-0893. PubMed: 18203673. 18203673 PMC4091824

[33] Huang Z, Rivas B, Agoulnik AI (2012) Insulin-like 3 signaling is important for testicular descent but dispensable for spermatogenesis and germ cell survival in adult mice. Biol Reprod 87: 143. doi:10.1095/biolreprod.112.103382. PubMed: 23100620. 23100620 PMC4435430

